# Community-driven approach to promoting healthy culturally tailored diets and cardiometabolic health among East African immigrants in San Diego: a community case study

**DOI:** 10.3389/fpubh.2026.1788981

**Published:** 2026-07-20

**Authors:** Nicole N. Karongo, Xochitl Aguinaga, Amy E. Atun, Sahra Abdi, Tsigealem Birhane, Meshate Mengistu, Connie Weaver, Cheryl A. M. Anderson

**Affiliations:** 1 Herbert Wertheim School of Public Health and Human Longevity Science, University of California, San Diego, La Jolla, CA, United States; 2 School of Public Health, San Diego State University, San Diego, CA, United States; 3 United Women of East Africa Support Team, San Diego, CA, United States; 4 School of Exercise and Nutritional Sciences, San Diego State University, San Diego, CA, United States; 5 Division of Nephrology and Hypertension, Department of Medicine, University of California, San Diego, La Jolla, CA, United States

**Keywords:** capacity building, Community Based Participatory Research (CBPR), community-driven research, culturally tailored nutrition interventions, East African immigrants, health equity, social participation

## Abstract

The East African immigrant community in San Diego has proactively sought multi-generational, nutrition-focused strategies for the prevention and management of cardiometabolic disease. Existing studies on Somali immigrants in the U.S. Midwest document elevated rates of diabetes (4.5–21.1%), overweight/obesity, and dyslipidemia, while qualitative evidence indicates that dietary acculturation, particularly within the first decade of migration, is associated with decreased consumption of traditional foods and increased exposure to sodium- and sugar-dense processed foods. This community case study describes the long-standing community-academic partnership between United Women of East Africa Support Team (UWEAST) and the University of California, San Diego Herbert Wertheim School of Public Health Anderson Lab, operationalized through the Healthy Eating with Spices and Herbs to Manage Hypertension (HAWAASH) research project series and formative phase of HAWAASH2. Guided by Community Based Participatory Research (CBPR) and community-driven research principles, the partnership prioritized social participation, empowerment, and capacity building through shared governance, community-led recruitment, multilingual engagement strategies, and culturally responsive implementation practices. Using a convenience sampling approach with an iterative mixed-methods formative design, 60 community members and seven key informants contributed to shaping the design of a future culturally tailored nutrition intervention. Activities were conducted at the UWEAST community hub in City Heights, San Diego. Grounded theory-guided thematic analysis identified five themes from community member interviews and four themes from key informant interviews, encompassing cultural food identity, structural barriers to healthy eating, dietary acculturation experiences, community social assets, and readiness for nutrition programming. This case study highlights practical strategies, lessons learned, and contextual constraints relevant to advancing equitable, community-driven nutrition initiatives among underrepresented immigrant populations. Detailed findings on measurement tool acceptability and proof-of-concept intervention plausibility are reported in companion manuscripts.

## 1 Introduction

Health equity efforts increasingly recognize that addressing disparities requires strengthening participation, empowerment, and capacity building within communities most affected by inequitable health outcomes. East African immigrants in the United States are a growing yet underrepresented population in cardiometabolic health research. Structural barriers to food access, dietary acculturation, and competing social demands contribute to elevated cardiometabolic risk over time, underscoring the urgency of community-driven, culturally grounded intervention approaches.

East African immigrants account for 38% of Sub-Saharan African immigrants living in the U.S., yet their experiences are substantially underrepresented in nutrition and public health research. Within California, an estimated 149,467 people identify as Sub-Saharan African immigrants, with 44% from East Africa. San Diego holds the second largest population of East African immigrants in California, and San Diego County ranks 7th nationally for the highest numbers of Somali and Sudanese immigrants (1). Despite this growing presence, very few studies characterize the cardiometabolic health profile or dietary behaviors of this population. Studies that do exist, primarily from the Midwest, document elevated rates of diabetes and obesity among Somali immigrants compared to national averages and qualitative research consistently identifies dietary acculturation, particularly increased consumption of fast food and decreased use of traditional herbs, spices, and whole foods, as a key driver of worsening health over time (2–6).

Community Based Participatory Research (CBPR) is defined as “a collaborative research approach that is designed to ensure and establish structures for participation by communities affected by the issue being studied, representatives of organizations, and researchers in all aspects of the research process to improve health and wellbeing through taking action, including social change” (7). Over the past two decades, CBPR has been increasingly adopted in public health research to foster trust, improve recruitment and retention, and produce more contextually relevant findings (8, 9). The Community-Driven Research Framework (CDRF) extends these principles by centering the community as the primary driver of inquiry (10). Among East African immigrant communities specifically, CBPR approaches have supported improved recruitment, retention, and dissemination compared to traditional investigator-driven studies (2, 3). Cultural assets such as social cohesion, interconnectedness, and interpersonal networks, particularly among Somali and Oromo communities, can be leveraged to support health promotion initiatives (2, 11). Both CBPR and CDRF are rooted in the premise that health inequities are best addressed through democratic partnerships that elevate lived experience alongside academic expertise.

Traditional dietary patterns among East African immigrants are often disrupted during acculturation, particularly within the first decade of migration, due to structural and social barriers to accessing familiar foods (4–6). This disruption may contribute to nutritional inequities and increased cardiometabolic risk. Culturally tailored dietary interventions have been shown to improve acceptability and sustainability by aligning health recommendations with cultural values and food practices (12). The Healthy Eating with Spices and Herbs to Manage Hypertension (HAWAASH) research program was developed to examine cultural influences on dietary behaviors and cardiometabolic health among East African immigrants and to inform the development of culturally responsive nutrition interventions (13). This community case study describes how a long-standing community-academic partnership operationalized participation, empowerment, and capacity building to inform the design of a culturally tailored nutrition intervention for East African immigrants living in San Diego. A framework for understanding dietary acculturation in this population, findings from the formative qualitative research conducted in this case study informed the design of subsequent co-design workshops and a proof-of-concept cooking intervention will be published in subsequent manuscripts.

## 2 Context

### 2.1 Community setting and partnership history

United Women of East Africa Support Team (UWEAST) is a community-based organization established in 2009 that serves East African immigrant communities throughout San Diego County. UWEAST operates through a participatory organizational model in which decisions are made through group consensus and programs are facilitated largely by community members. Over time, UWEAST has developed capacity in translation services, research support, peer education, and community-based programming. The organization serves over 720 community members, most of whom live within 15 miles of the organization's City Heights location.

The community-academic partnership between UWEAST and the University of California, San Diego Anderson Lab emerged in 2014 through ongoing collaboration on community-identified health priorities. Nutrition and cardiometabolic health were identified by UWEAST members as areas of concern, prompting the development of the first HAWAASH study in 2015 (13). This qualitative study explored dietary acculturation and nutrition transition among East African immigrants and identified both cultural strengths, including robust home-cooking practices, knowledge of food-growing methods, and strong interest in nutrition education, and barriers, including time constraints, rising food costs, and increased exposure to fast food. Participants highlighted a need for collaborative educational programs and cooking classes that promote healthy eating across culturally heterogeneous African communities in the United States. UWEAST affirmed the value of continuing the community–academic partnership, noting that the 2015 HAWAASH study fostered open and supportive dialogue around nutrition and created a trusted space for community members to engage in health-related discussions (13). Community partners emphasized the importance of collaborating with an academic institution to pursue nutrition and health research aligned with community priorities, including interventions aimed at reducing cardiometabolic disease risk. Through subsequent discussions between partners, it was collectively identified that there was a need to deepen understanding of nutrition-related decision-making within the community, particularly in relation to dietary acculturation experiences.

### 2.2 Population and social-structural context

**Figure 1:**
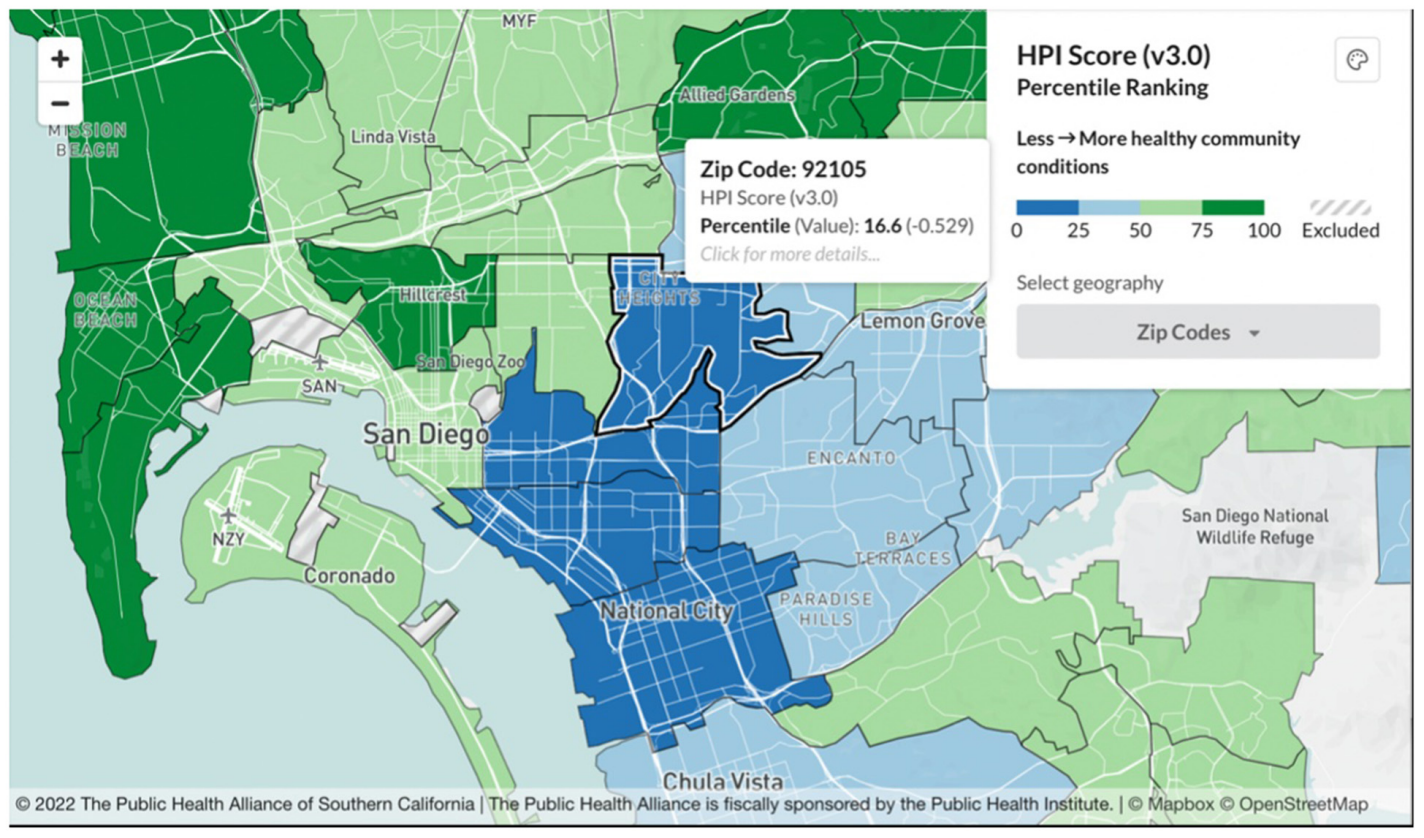
Healthy Places Index (HPI) score of the City Heights community (zip code 92105).

**Figure 2:**
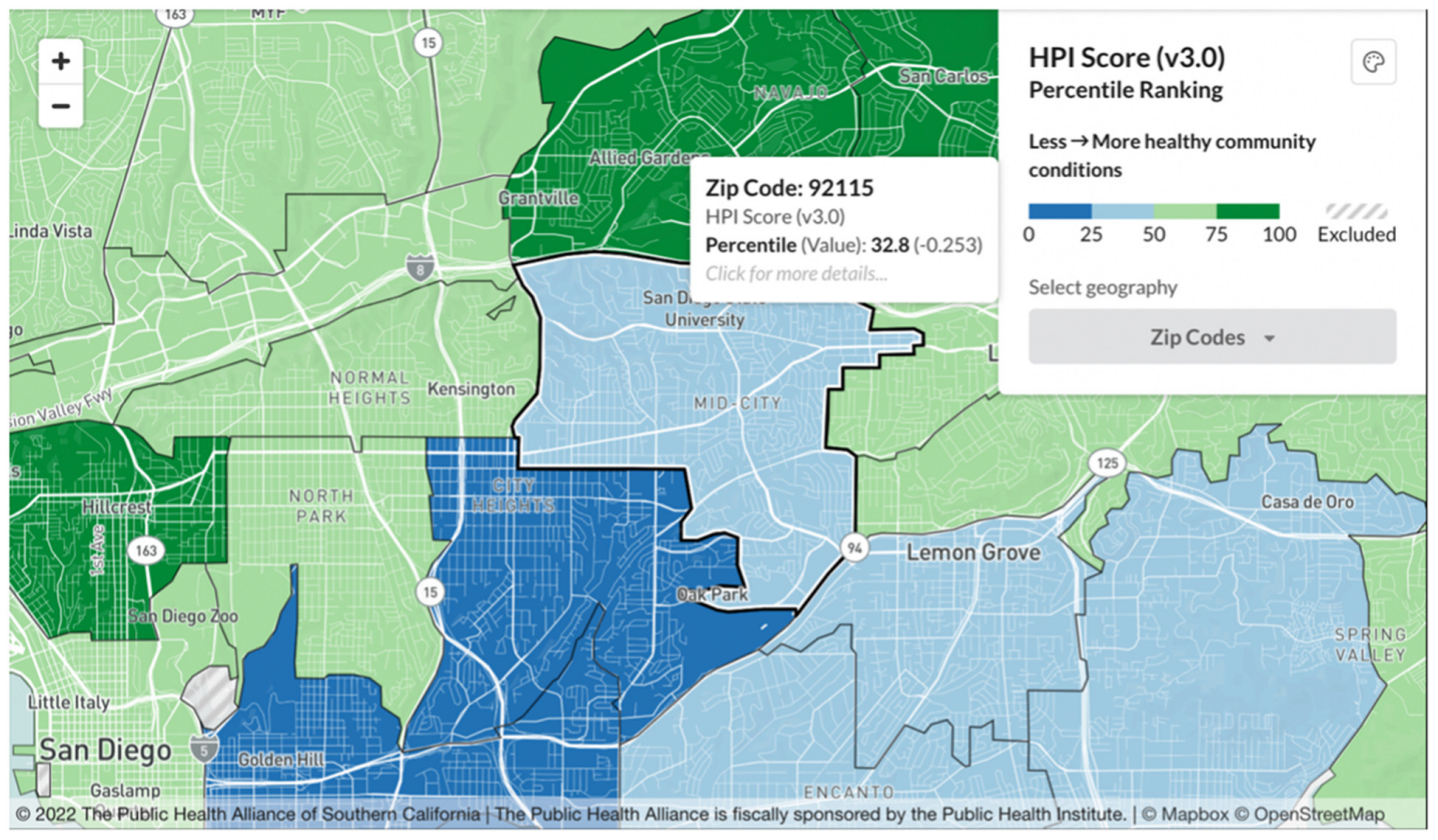
Healthy Places Index (HPI) score of the Mid-City community (zip code 92115).

HAWAASH2 continued its focus on East African immigrant adults engaged through UWEAST and partner organizations. Approximately 75% of participants resided in the City Heights (92105) or Mid-City (92115) neighborhoods of San Diego. These areas have Healthy Places Index (HPI) scores of 16.6 and 32.8, respectively, both below the county average of 67.9, reflecting structural challenges related to economic conditions, housing, and healthcare access [the HPI scale ranges from 0 [least favorable] to 100 [most favorable]] (14). Table 1 presents HPI scores by participant zip code. Figures 1, 2 show the HPI score for City Heights (92105), and Mid-City (92115), respectively. Such social-structural conditions are known contributors to cardiometabolic risk and shape both the feasibility and acceptability of health interventions (15, 16). Limited access to culturally competent healthcare, distrust of healthcare systems, and cost barriers further compound health inequities among African immigrant populations (17, 18). These contextual factors highlight the importance of community-driven, cost-conscious preventive strategies that are grounded in the lived realities of the population.

**Table 1:** HPI scores by zip codes of participants.

Zip code	Number of participants residing	HPI score (percentile)
92105	25	16.6
92115	20	32.8
92114	4	33.8
91945	3	37.9
92126	2	73.4
91942	1	58.7
91978	1	58.9
92120	1	82.4
92104	1	65.3
92119	1	83.3
92139	1	42.9

## 3 Methodology

### 3.1 Research design and theoretical framework

HAWAASH2 was informed by the CBPR conceptual model developed by the University of New Mexico Center for Participatory Research (8, 9, 19). The model includes four dynamic domains: (i) Research design, (ii) contextual factors, (iii) partnership processes, and (iv) outcomes. Each domain informs and influences the others, providing a framework for evaluating participatory practices and their impact on equity-related outcomes. The formative phase of HAWAASH2 was designed to address the co-created research question: “What strategies and approaches are helpful in addressing community needs to achieve impactful and sustainable nutrition-related health behaviors with the East African immigrant community of San Diego?” An iterative mixed-methods approach was used to integrate perspectives from community members and key informants in the design of a future culturally tailored nutrition intervention.

This case study is descriptive in nature, reporting on the operational and relational elements of the community-academic partnership and the formative qualitative findings that informed subsequent phases of the research. Evaluative components, specifically the assessment of measurement tool acceptability and intervention plausibility, are reported in companion manuscripts.

### 3.2 Setting

All group interviews and partnership activities were conducted at the UWEAST community hub located in the City Heights neighborhood of San Diego (zip code 92105). This location was selected because it is the central gathering space for UWEAST members, most of whom reside within a 5-mile radius of the organization. The UWEAST hub functions as a trusted, culturally safe space where community members regularly participate in programs, prayer, childcare, and community events, making it an ideal setting for research engagement.

### 3.3 Sampling strategy and participants

Recruitment and data collection were limited to UWEAST and partner organization sites (the Refugee Assistance Center and the South Sudanese Center) to leverage existing trust and organizational reach. A convenience sampling approach was used, which was supplemented by purposive recruitment targeting individuals with experience of dietary acculturation, elevated cardiometabolic risk, and engagement with community programming. Two UWEAST staff members led recruitment through internal outreach, social media posts (Instagram @uweast1), and pre-existing UWEAST WhatsApp groups. This approach allowed for resource efficiency while maintaining community reach.

Participants were screened through a two-step process: a self-administered REDCap survey with multilingual technical assistance, followed by in-person intake sessions with informed consent. Eligibility criteria included being 18 years or older and having hypertension, pre-hypertension, or a family history of hypertension. Our sampling efforts allowed us to recruit 60 community members who identify as East African immigrants to serve as study participants. Of these, 48 participated in group interviews. Seven key informants were additionally recruited based on their professional and leadership roles serving the East African immigrant community in San Diego. Key informants held multi-level personal and professional roles in the East African community in San Diego including positions as grassroots community leaders, community health workers, mental health specialists, program directors, and program coordinators. All key informants identified as East African, with countries of origin including Ethiopia and Somalia.

### 3.4 Group interviews and key informant interviews

Semi-structured group interviews with 48 community members explored experiences of dietary acculturation, community wellness, dietary behaviors, confidence, and interest in future nutrition programming. Groups were matched by language and gender to facilitate open dialogue. Groups consisted of a maximum of five community members at a time to promote participation and reduce interpretation challenges. An interpreter (Somali, Amharic, or Arabic) was provided for each non-English group. Key informant interviews were conducted with seven community leaders via Zoom or in-person at UWEAST, lasting 45-60 minutes each. Questions addressed barriers and challenges to healthy eating, program design recommendations, and community strengths and opportunities. Worksheet aids were used during group interviews to support structured ranking and brainstorming tasks. See Supplementary Table S1 for full interview questions for each protocol.

### 3.5 Data analysis

Quantitative data were analyzed using R (version 2023.12.0+369) (Posit Software, PBC, Boston, United States) to generate descriptive statistics characterizing the sample. Qualitative data analysis was performed using NVivo 12 (Posit Software, PBC, Boston, United States). Group interview transcripts and corresponding field notes were analyzed using grounded theory-guided thematic analysis in three phases: open coding, focused coding, and selective coding (20). In open coding, two senior researchers or one senior and two junior researchers independently reviewed transcripts and generated codes directly from the data. Focused coding sessions brought researchers together to identify emerging categories and themes from across open codes. Selective coding identified the most prominent themes across all data. Key informant interview transcripts were analyzed similarly, with one senior researcher conducting open and focused coding organized around the 12 interview questions, followed by selective coding to identify cross-cutting themes.

### 3.6 Partnership governance and shared decision making

Over the 10 years of partnership between UWEAST and the UC San Diego Anderson Lab, relationships with the community were maintained through ongoing discussions and meetings. Roles and responsibilities were formalized through a Memorandum of Understanding (MOU), renewed annually over the 2-year project period. The MOU outlined shared goals, ethical considerations, and division of resources. Table 2 summarizes the distribution of roles and responsibilities.

**Table 2:** Distribution of roles and responsibilities.

UWEAST role and responsibilities	Anderson lab role and responsibilities
Study recruitment	Scientific expertise and study design
Participant communications	Administration of study activities
Translation & interpretation services	IRB and regulatory processing
Site for study activities	Participant compensation
Site for free childcare	Data management and analysis
Catering services (Baraka Bilal)	

### 3.7 Community-led recruitment and engagement

United Women of East Africa Support Team staff led all recruitment activities to leverage existing trust within the community. Recruitment was streamlined through UWEAST-managed communications, which contributed to an 85% retention rate over the 2-year study period. Language access was prioritized through subcontracting multilingual community members to provide interpretation services in Somali, Amharic, Arabic, and Swahili. All group interviews were conducted at the UWEAST hub in a trusted community space. Childcare services were provided by UWEAST staff during study activities to reduce participation barriers, particularly for mothers. Food was provided through UWEAST's community catering enterprise, Baraka Bilal, which employs community members to prepare traditional East African dishes, reinforcing cultural relevance while supporting community economic participation.

### 3.8 Reflexivity, positionality, and capacity building

Reflexivity, defined by Dowling et al. as “the analytic attention to the researcher's role in qualitative research,” and positionality were intentionally and explicitly addressed throughout the partnership (21–23). UWEAST specifically requested collaboration with researchers who also identify as immigrants, reflecting community values around shared lived experience, cultural understanding, and trust. In response, the research team was led by Afro-Caribbean and African immigrant researchers, which facilitated rapport building, mutual respect, and more open dialogue during recruitment, data collection, and interpretation.

All UWEAST staff and leadership identified as East African and brought their lived experiences into the project in ways that actively shaped study implementation. Community members who served as interpreters, caterers, and childcare providers were compensated competitively, functioning as a mechanism to recognize community expertise, redistribute resources, and support organizational and individual capacity. Together, these practices operationalized participation, empowerment, and capacity building as interrelated mechanisms within the community-academic partnership.

## 4 Findings

### 4.1 Participant characteristics

Among the 63 community members enrolled, the sample was 83% female with a median age of 42 years (IQR: 27–54). Most participants (78%) were born outside the United States. The sample represented diverse East African countries of origin including Somalia (38%), Sudan (14%), Ethiopia (14%), Eritrea, Kenya, and Morocco. Ninety percent identified as Muslim. Forty-eight percent utilized interpretation services. Sixty-five percent held a high school diploma or less, and 81% reported household incomes below the San Diego median of $98,657. Most participants (90%) lived within a 5-mile radius of UWEAST.

Clinically, the sample had a mean systolic blood pressure of 116.5 mmHg (SD = 12.6) and a mean diastolic blood pressure of 75.8 mmHg (SD = 9.2). The mean BMI was 29.1 kg/m^2^ (SD = 7.3). Over one third of participants (35%) reported a hypertension diagnosis. Of those without a diagnosis (*n* = 41), 85% reported a family history of hypertension. Twenty-seven percent reported having diabetes, which is higher than both the U.S. national prevalence (15.8%) and the African American prevalence (14.6%) of diabetes. When asked about post-migration health compared to pre-migration health, 41% reported that their health had worsened since moving to the United States. These clinical characteristics underscore the urgency of culturally responsive cardiometabolic prevention efforts for this population.

Dietary and lifestyle behaviors indicated that 75% of community members cooked at home always or often, and 63% always or often incorporated cultural traditions when making food choices. Eighty-two percent reported using more than 10 distinct spices or herbs at least once in the month prior to data collection, reflecting the centrality of herbs and spices in East African cooking. These findings reinforce the potential of herbs and spices as a culturally grounded entry point for nutrition education and intervention.

### 4.2 Qualitative themes from community members

Five overarching themes emerged from grounded theory-guided thematic analysis of group interview data. Table 3 summarizes these themes with illustrative participant quotes.

**Table 3:** Themes from group interviews with community members (*N* = 48).

Theme	Description	Illustrative quote
1. Home country food is perceived as superior	Participants described home country foods as fresher, more organic, and more flavorful than U.S. foods. Trust in U.S. food was low due to concerns about preservatives and freezing.	“*Mostly we have organic food back home and everything is natural, daily, there's no processed, but here we eat it processed. That's the difference I'm feeling.”* (GI 2, HSV026)
2. Dietary patterns have changed since migration	Community members linked shifts in food environment to worsened cardiometabolic health. Greater food variety, refrigerated foods, and fast food exposure were identified as drivers of dietary change.	“*I believe that everything is different from what we eat right now. Because I used to eat fresh food every morning. And there, nobody has diabetes or high blood pressure or anything.”* (GI 15, HSV029)
3. Cultural food practices are protective	Participants expressed desire to maintain traditional cooking using herbs and spices. Cultural identity was closely tied to food preparation and mealtimes as communal practices.	“*Our spices are medicine. When I cook with cardamom and ginger like my mother taught me, I feel strong.”* (GI 7)
4. Structural barriers limit healthy eating	Participants identified multilevel barriers including cost of healthy food, limited time for cooking due to work demands, and children's preference for non-traditional foods.	“*Everything is expensive. The healthy food is very expensive so I have to sometimes get what my kids want which is not what I cooked growing up.”* (GI 11)
5. Community readiness for nutrition programming	Participants expressed strong interest in culturally tailored cooking classes, recipe exchanges, and peer-based nutrition education. Social cohesion was identified as a facilitator for group programming.	“*If we could come together and cook and learn together, that would help all of us. We learn better when we see and do.”* (GI 3)

Across themes, several multilevel barriers to healthy eating were identified. At the individual level, participants described shifts in dietary choices and reduced familiarity with U.S. food labeling. At the relational level, tensions around children's preferences for non-traditional foods and limited household help with food preparation were noted. At the community level, participants described reduced access to familiar ingredients and fewer social systems for sharing food resources. At the societal level, participants noted that the pace and structure of life in the United States, including long working hours and transportation demands, limited time for traditional cooking.

These multilevel findings are consistent with the DANIA (Dietary Acculturation of African Immigrants in the U.S.) Framework (subsequent manuscript), which identifies individual, relational, community, and structural factors as interconnected determinants of dietary behavior change among African immigrants.

### 4.3 Key informant characteristics

Four themes emerged from key informant interviews. Table 4 presents these themes and illustrative quotes.

**Table 4:** Themes for key informant interviews.

Theme	Description	Illustrative quote^*^
1. Structural and systemic barriers to healthy eating	Key informants identified economic hardship, food desert conditions in City Heights, and limited access to culturally familiar produce as key barriers. Healthcare access and language barriers compounded these challenges.	“*I think those are the main challenge, for not to have access for good food or healthy food. Finance and transportation, not knowing where to go, and also how to ask for support when they need it.”* (HKID001)
2. Gender and social norms shape food procurement and nutrition programming engagement	Food preparation is traditionally women's domain. Male engagement in nutrition programming is limited by cultural norms. Informants recommended gender-specific, peer-led programming for males.	“*I think definitely we need to have more male participants involved in the cooking.”* (HKID004)
3. Community strengths can be leveraged	Strong social networks, religious community cohesion, and WhatsApp-based communication were identified as assets for disseminating health information and supporting intervention uptake.	“*… workshops have worked really well, because the community really loves being together and like having group things, they're always really popular and gets people out of the house.”* (HKID007)
4. Nutrition programs must be culturally grounded and cost-conscious	Informants emphasized that future interventions must honor traditional East African dietary practices, be low-cost, and avoid stigmatizing community food behaviors. Programs should center community expertise.	“*… not trying to change their culture, because that's what they grow up eating. But to kind of also give them that ‘we could make that healthy', we can still enjoy the food and still kinda make it healthier.”* (HKID006)

^*^All quotes are reproduced verbatim from individual interview transcripts. Minor omissions are indicated with ellipses (…). HKID, HAWAASH2 Key Informant ID. Quotes represent four of seven key informants (HKID001, HKID004, HKID006, HKID007). The remaining three informants (HKID002, HKID003, HKID005) expressed views consistent with the themes presented; their responses were not quoted here due to thematic redundancy.

Key informants provided critical contextual intelligence that community member interviews alone could not capture, including insights on institutional barriers, gender dynamics, and strategies for sustainable program design. Their perspectives affirmed that any future nutrition intervention must be cost-conscious, gender-sensitive, faith-aware, and community-led to be effective and trusted.

### 4.4 How interview findings inform intervention design

The themes generated from community member and key informant interviews directly shape the design of subsequent phases of the HAWAASH2 research program. First, the centrality of herbs and spices in community cooking (Theme 3) confirmed herbs and spices as the primary culturally resonant vehicle for delivering nutrition education on sodium, added sugar, and saturated fat reduction. Second, the multilevel structural barriers identified (Theme 4) informed a cost-conscious intervention design, with all activities hosted at UWEAST and food provided through Baraka Bilal to eliminate cost barriers for participants. Third, strong expressed interest in peer learning and communal cooking (Theme 5) led the research team to prioritize group-based cooking classes over individual counseling as the primary intervention modality.

Fourth, key informant insights regarding gender norms (KI Theme 2) led to the development of two gender-specific proof-of-concept trial groups, a dietitian-led group of older foreign-born women (Group 1) and a peer-led group of younger U.S.-born men (Group 2), to test delivery modalities appropriate to each subgroup. Fifth, key informant recommendations for halal-compliant and culturally familiar food (KI Theme 4) ensured that all food samples and cooking class recipes incorporated traditional East African ingredients and were halal-certified.

Consistent with CBPR principles, intervention materials will be co-developed with the community. All recipes, curriculum content, and educational materials are reviewed and adapted in partnership with UWEAST staff. Interpreters from within the community participate in session facilitation to ensure linguistic and cultural accuracy. Future iterations will incorporate input from male community leaders and youth-facing programs to improve reach across demographic subgroups.

## 5 Discussion

### 5.1 Strengths of HAWAASH2 practices in advancing equitable research

This community case study demonstrates how a long-standing community-driven partnership can operationalize participation and capacity building in equitable nutrition research. Comparable CBPR-informed, co-design approaches have recently been applied in immigrant Hispanic/Latine communities with promising cardiometabolic outcomes, situating HAWAASH2 within a growing body of practice-based evidence (24).

A distinguishing feature of HAWAASH2 was the integration of UWEAST's organizational infrastructure into all aspects of the research, from recruitment and interpretation to catering and childcare. Our approach aligns with growing evidence that CBPR-informed interventions improve not only health outcomes but also engagement, retention, and research relevance for minoritized populations (21, 25, 26). The 85% retention rate observed over the 2-year study period reflects the strength of the UWEAST-Anderson Lab partnership and the trust-building strategies embedded in the study design. By embedding research activities within the community organization, we minimized logistical barriers for participants while reinforcing UWEAST's organizational capacity and visibility.

The multilingual engagement strategy, including interpretation in Somali, Amharic, Arabic, and Swahili, and the community interpreter model, helped facilitate genuine data collection among linguistically diverse participants. Participants shared more openly in their native languages, and the interpreters themselves (community members who were compensated for their skills) gained professional development and visibility.

### 5.2 Conceptual and methodological constraints

While CBPR is the desired mechanism for creating strong community-academic partnerships, researchers still face logistical and structural barriers to implementing this work in systems not designed to promote community-driven research (27). Restrictive institutional factors can slow the pace of trust-based relationships and create misalignments between academic timelines and community rhythms. It is therefore important to be aware of potential impediments and structure research programs to set clear expectations with community partners from the outset.

A key challenge of this study was balancing participant burden reduction, a core CBPR value, with scientific rigor. For example, 24-h dietary recall sessions required interpretation and often took over an hour to complete, causing frustration among participants. As a result, this method was suspended. Similarly, the Herb and Spice Questionnaire (HSQ) required extensive adaptation to reflect regional variation in herb naming, preparation forms, and measurement practices. Despite these refinements, the HSQ remained more time-intensive than anticipated. These experiences directly inform the selection and adaptation of measurement tools evaluated in the co-design workshops reported in a subsequent manuscript.

Another limitation was the low participation rate among male community members, which reflects cultural norms around gender and nutrition programming. Given that UWEAST's programming is primarily organized by women and for women, and that the research and outreach staff was primarily composed of women, male participants were less likely to be recruited.

Limitations related to generalizability should also be noted. The convenience sampling strategy and geographic concentration of participants in City Heights may limit the transferability of findings to East African immigrant communities in other cities or states. While the dominant pattern across interviews reflected strong cultural pride in traditional foodways and readiness for programming, a small number of participants noted that even traditional ingredients such as spices and specialty grains had become cost-prohibitive in the United States, representing a divergent view not fully captured in the main themes. Social desirability bias may have led to underreporting of barriers, as participants tended to minimize challenges when directly asked. Researchers observed contradictory statements between direct responses about barriers and incidental disclosures during interviews, consistent with the literature. Future studies should consider structured observational methods or validated barrier assessment tools to complement self-reported data.

### 5.3 Implications for practice, policy, and future research

This case study has several implications for public health practice. Community-based organizations like UWEAST are essential infrastructure for equitable health research and programming. Their existing trust, reach, and cultural fluency cannot be replicated by academic institutions alone. Future nutrition interventions for immigrant communities should be designed in partnership with such organizations from inception, not as implementation partners added after the research design is finalized, consistent with recommendations from Commodore-Mensah et al. (18).

For policy, findings highlight the need for federal and state nutrition programs, including WIC, SNAP, and SNAP-Ed, to be culturally responsive to East African and other immigrant populations, incorporating traditional foodways such as herbs and spices, legumes, and whole grain staples rather than defaulting to Western-normative dietary models. This is now reinforced at the federal level: the 2025 Dietary Guidelines Advisory Committee Scientific Report included the first-ever evidence scan on culturally responsive dietary interventions, concluding that culturally tailored approaches may improve adherence to dietary guidance and recommending dedicated funding for culturally responsive research and program evaluation (28). Policies that additionally fund bilingual staffing and culturally tailored educational materials would substantially strengthen the equitable reach of these programs, particularly for populations like ours in which 81% of participants earned below the San Diego household median and resided in neighborhoods with HPI scores in the bottom third of California. Ogungbe et al. similarly found that lower income, lack of health insurance, and limited social support were independently associated with hypertension and diabetes among African immigrants, reinforcing the need for structural determinants to be addressed alongside behavioral components in future intervention designs (29).

For future research, the HAWAASH2 program is well-positioned to advance toward a potential effectiveness trial integrating the tailored measurement protocols and cooking intervention curriculums. The recent Afro-DPP pilot cluster-randomized trial, the first published RCT of a culturally adapted lifestyle intervention for African immigrants in the U.S., reported systolic blood pressure reductions of 9.2–11.4 mmHg and diastolic reductions of 6.1–10.3 mmHg at 6 months, providing meaningful evidence that culturally grounded behavioral interventions are efficacious in this population and strengthening the rationale for advancing our work to a similarly rigorous design (30). Future studies should also more systematically examine associations between dietary behaviors, herb and spice use, and biomarker outcomes such as blood pressure, HbA1c, and 24-h sodium excretion, a gap that remains essentially unaddressed in the East African immigrant literature. Finally, greater attention to male engagement in nutrition research is warranted, and future work should explore peer-led, male-facilitated programming as an avenue for reaching this underserved subgroup.

## 6 Conclusion

We provide insights on promoting mutual trust and respect for lived experiences, which is a critical factor in developing culturally tailored dietary interventions for optimal cardiometabolic health. By developing interventions that are informed and designed with community insights at the helm, historically marginalized populations, such as U.S.-based East African immigrants, will be better served through sustainable research and programmatic efforts. As the field of public health advances toward community-driven approaches, preservation of trust between academic partners and the community members they wish to serve should be at the forefront from design to dissemination.

## Data Availability

The raw data supporting the conclusions of this article will be made available by the authors, without undue reservation.
