# Supplementary material for: Community-driven approach to promoting healthy culturally tailored diets and cardiometabolic health among East African immigrants in San Diego: a community case study
Source: Front Public Health. 2026 Jul 20;14:1788981. doi: 10.3389/fpubh.2026.1788981 (PMC13429831; doi:10.3389/fpubh.2026.1788981)
Supplement: Supplementary file 1 [file Table_1.docx]

**Supplementary Materials**

**Table S1. Interview Questions for Community Member Group Interviews and Key Informant Interviews**

| **Foreign-Born Community Member Group Interview Questions (N=48, foreign-born subset)** | **U.S.-Born Community Member Group Interview Questions (U.S.-born subset)** | **Key Informant Interview Questions (N=7)** |
| --- | --- | --- |
| 1. Can you tell me what country are you from and how long you have been in the United States? | 1. What country/region is your family from and how many generations has your family lived in the United States? | 1. Tell me your name and your title and organization (if applicable)? |
| 2. [Worksheet Aid] When you think of healthy and unhealthy foods for blood pressure, what comes to mind for:  • Individual foods  • Prepared dishes  • Individual ingredients (including herbs, spices, food additives) | 2. [Worksheet Aid] When you think of healthy and unhealthy foods for blood pressure, what comes to mind for:  • Individual foods  • Prepared dishes  • Individual ingredients (including herbs, spices, food additives) | 2. Can you briefly, in 1–2 sentences, tell me about your involvement in the East African community in San Diego? |
| 3. [Worksheet Aid] On a scale of 1–10, how would you compare the foods you now eat in the United States vs. the foods you normally ate in your home country? (10 = completely different; 1 = exactly the same)  • Score 1–4: Why is your diet similar?  • Score 5–10: What caused your diet to change so much? | 3. [Worksheet Aid] On a scale of 1–10, how would you compare the foods you now eat in the United States vs. the foods you might eat in the country you culturally identify with? (10 = completely different; 1 = exactly the same)  • Score 1–4: Why would your diet be similar?  • Score 5–10: Why would your diet be different? | 3. Can you briefly tell us what types of activities have you been involved in within the community, whether personally, professionally, or on behalf of your organization? |
| 4. [Worksheet Aid] Please rank the following food choice priorities from 1 (most important) to 7 (least important), excluding religious considerations, allergies, or intolerances: cultural familiarity, healthfulness, household preferences, preparation, price and budget, storage capacity, taste. | 4. [Worksheet Aid] Please rank the following food choice priorities from 1 (most important) to 7 (least important), excluding religious considerations, allergies, or intolerances: cultural familiarity, healthfulness, household preferences, preparation, price and budget, storage capacity, taste. | 4. Which portion of the community, if any, have your efforts been focused on? Who have you reached or have access to? |
| 5. By a show of hands, do you feel that your health has changed since coming to the United States?  • If yes: In your view, what is the biggest contributor to that change? | 5. What are your specific concerns about your health when it comes to the kinds of food you eat? (Where do those concerns come from: bodily reaction or information source?) | 5. What special challenges do you think would be faced when trying to create a program about healthy eating in the East African community? |
| 6. What are your specific concerns about your health when it comes to the kinds of food you eat in the United States? | 6. Some people feel that preparing healthy home-cooked meals can be stressful. What are some of the challenges you face when preparing healthy meals at home? | 6. What suggestions do you have in terms of how we might meet these challenges? |
| 7. Some people feel that preparing healthy home-cooked meals can be stressful. What are some of the challenges you face when preparing healthy meals at home? | 7. What would make it easier for you to prepare healthy meals at home? | 7. What do you think are the keys to successfully creating a healthy eating program targeted to the East African community in San Diego? Why do you feel that way? |
| 8. What would make it easier for you to prepare healthy meals at home? | 8. How much salt do you use to flavor food? | 8. What do you think are the greatest obstacles to successfully improving dietary health among this community? Why do you feel that way? |
| 9. How do you use salt to flavor food? (What are the different ways you use salt?) | 9. How do you use spices and herbs to flavor food? Why do you choose to use spices?  • Are there spices and herbs you are interested in trying but have not used yet? If yes, why not? | 9. Do you have any thoughts or ideas about programs, strategies, partners, or activities that you think would be particularly effective for communicating healthy eating to the East African community? |
| 10. How do you use spices and herbs to flavor food? Why do you choose to use spices?  • Are there spices and herbs you are interested in trying but have not used yet? If yes, why not? | 10. [Worksheet Aid] We are designing a program where you learn about nutrition and try new recipes. What would you want to see in that program? Please use the worksheet to list the top 5 things you would like to see in terms of recipes, ingredients, dishes, and nutrition topics. | 10. If you could design a 2-year program to improve healthy eating outcomes among San Diego East Africans, what would you do? What do you think are the most essential components? |
| 11. [Worksheet Aid] We are designing a program where you learn about nutrition and try new recipes. What would you want to see in that program? Please use the worksheet to list the top 5 things you would like to see in terms of recipes, ingredients, dishes, and nutrition topics. |  | 11. What if we taught group cooking classes focused on using community strengths such as cooking skills and spice use, and adapted these to meet nutrition recommendations? What do you think of this idea? What would you add to it? |
|  |  | 12. Is there anyone else in the community I should speak with about healthy eating strategies, programs, and activities that could help improve healthy eating outcomes in the East African community? |

* *Note. Worksheet aids consisted of printed handouts used to support structured ranking and brainstorming tasks during group interviews. KI = Key Informant. U.S.-Born protocol was adapted from the Foreign-Born protocol to reflect generational differences in cultural food identity.*
